# All Components of Metabolic Syndrome Are Associated with Microalbuminuria in a Chinese Population

**DOI:** 10.1371/journal.pone.0157303

**Published:** 2016-06-21

**Authors:** Yi-Yen Lee, Chih-Kai Yang, Yi-Ming Weng, Chung-Hsun Chuang, Wei Yu, Jih-Chang Chen, Wen-Cheng Li

**Affiliations:** 1 Division of Pediatric Neurosurgery, Neurological Institute, Taipei Veterans General Hospital, Taipei, Taiwan; 2 Faculty of Medicine, National Yang-Ming University, Taipei, Taiwan; 3 Department of Emergency Medicine, Xiamen Chang-Gung Hospital, Xiamen, China; 4 Department of Emergency Medicine, Chang-Gung Memorial Hospital at Linkou, Taoyuan, Taiwan; 5 Department of Health Management, Xiamen Chang-Gung Hospital, Xiamen, China; 6 Department of Medicine, College of Medicine, Chang-Gung University, Taoyuan, Taiwan; Shanghai Institute of Hypertension, CHINA

## Abstract

**Background and Aim:**

Albuminuria is a well-known predictor of poor renal and cardiovascular outcomes and associated with increased risk of all-cause mortality. The study aimed to evaluate the associations between metabolic characteristics and the presence of albuminuria.

**Methods:**

This cross-sectional study included 18,384 adult Chinese who participated in health examinations during 2013–2014. Differences in clinical characteristics were compared for microalbuminuria (MAU) and albuminuria, and between genders. Potential risk factors associated with the risk of developing MAU and albuminuria were analyzed using univariate logistic regression. Multiple logistic regression was applied to further identify the independent associations between different levels of risk factors and the presence of MAU and albuminuria. The area under the ROC curve (AUC) was used to determine the discriminatory ability of metabolic risk factors in detecting albuminuria.

**Results:**

There were significant gender differences in clinical characteristics according to albuminuria status. Risk for the presence of albuminuria was significantly associated with age, male gender, waist circumference (WC), waist-to-height ratio (WHtR), hypertension, fasting plasma glucose (FPG), and triglycerides to high-density lipoprotein cholesterol ratio (TG/HDL-C) in univariate logistic regression. Multiple logistic regression analysis indicated that the factors significantly associated with the presence of MAU were WC > 90cm, WHtR at 0.6–0.7, hypertension, FPG > 6.1 mmole/L, and TG/HDL-C ratio > 1.6. The optimal cutoffs for risk factors of metabolic syndrome (MetS) to predict albuminuria in males and females were: WC, 90.8 vs. 80.0 cm; WHtR, 0.53 vs. 0.52; MAP, 97.9 vs. 91.9 mmHg; FPG, 5.40 vs. 5.28 mmole/L; and TG/HDL-C, 1.13 vs. 1.08.

**Conclusion:**

MetS and all its components were associated with the presence of MAU in a health check-up population in China. Gender specific and optimal cutoffs for MetS components associated with the presence of MAU were determined.

## Introduction

Urinary albumin excretion is a predictor of mortality from all causes in the general population [1], and can be categorized into three stages: normoalbuminuria (<30 mg/24 h), microalbuminuria (30–300 mg/ 24 h) and macroalbuminuria (>300 mg/24 h). Emerging evidence indicates that microalbuminuria (MAU) is significantly and independently associated with risk for all-cause mortality, cardiovascular disease (CVD), chronic kidney diseases (CKD), and progression of end-stage renal disease (ESRD) [2–5]. Studies have also demonstrated that MAU is also a powerful predictor of CVD in both diabetic and non-diabetic subjects [6, 7]. MAU is usually absent at diagnosis but may develop in children with type 1 diabetes and poorer metabolic control [8]. On the other hand, intensive control of blood glucose level can prevent the onset and progression of MAU [9], and the incidence of decreased estimated glomerular filtration rate (eGFR) was rare in diabetic patients without preceding MAU [10].

MAU is commonly accompanied in the data concerning the interrelationship between metabolic syndrome (MetS), CVD, and CKD [11]. Specifically, there have been studies investigating the link between MAU and MetS [12–15]. Positive relationships between albuminuria and the prevalence of MetS and its components were shown in non-diabetic hypertensive individuals [16] and type 2 diabetes [17]. In the Third National Health and Nutrition Examination Survey (NHANES III), MAU was associated with MetS, and mainly with the fasting plasma glucose and blood pressure [12]. These observations enabled the published guidelines to include MAU as screening criteria for diabetic kidney disease [18].

The prevalence of MetS in the world is rapidly increasing [19], ranging from 9.8% to 46.5% in developing countries [20]. In mainland China, the prevalence was 15.1% in Chinese adults aged 35–74 years in a national cross-sectional study [21], but increased to 23.3% according to NCEP-ATPIII criteria [22, 23]. In addition, the prevalence of MAU was consistently higher (P < 0.0001 for all) in individuals with MetS than those without in Chinese (20.3% vs. 2.0%) [24], Japanese (20.8% vs. 12.2%) [15], and US Americans (13.7% vs. 4.8) [12].

To date, some studies have evaluated the association between MetS and MAU as a marker for early-stage CKD [12, 15, 25, 26]. Significant relationships between MetS and MAU have been demonstrated in Japanese [15] and Korean [25, 27], and in middle- aged and elderly Chinese population [28]. However, data concerning the relationship between individual MetS components and MAU were inconsistent [12, 15, 25], and it remains unclear the causal relationship between MAU and MetS in spite of the predictive value of MAU has been given in these well-established relationships as above-mentioned.

The aim of this cross-sectional study was to evaluate the association between metabolic characteristics and the presence of albuminuria, and to investigate the involvement of MetS risk factors for the presence of MAU in an adult Chinese population attending health examinations.

## Materials and Methods

### Subjects

We retrospectively collected the medical examination records of Chinese adults (aged ≥18 years) undergoing health check-ups during 2013–2014 at Health Examination Center, Xiamen Chang Gung Memorial Hospital, China. Subjects who met any of the following criteria were excluded from this study: (1) without a minimum of 12 hours of fasting prior to blood sampling; (2) pregnant women; (3) diagnosed with chronic diseases that significantly contribute to changes in metabolic functions, including thyroid disorders such as thyroid gland cancer and/or removal, chronic hepatitis, liver cirrhosis, hypothalamus disease, adrenal gland disorders; (4) with non-diabetic nephropathy. A total of 10,331 males and 8,053 females were included for analysis. The study was approved by the Institutional Review Board of Chang Gung Memorial Hospital and was conducted in accordance with the Helsinki Declaration.

### Data collection

Trained nurses were responsible for collecting information through interviews using a questionnaire consisted of questions on the following information: histories of past illness, medication, and physiological conditions (including pregnancy and fasting time) at the beginning of the examination. During the same visit, blood pressure (BP), height, weight, and waist circumference (WC) were measured and fasting blood samples were taken. All information was entered into a centralized electronic database under strict quality control monitoring at a regular basis. Data of patient records/information was anonymized and de-identified prior to analysis.

### Measurements of risk factors for CKD

Body Height and weight were measured using calibrated meters and scales. Body mass index (BMI) was calculated using the formula: body weight (Kg)/[(height in m)]^2^. To make sure the subject was not in an anxious status and confortable since physical stress (condition) can affect a BP reading, BP was measured three times after a 10-minute of rest, with the subject in the seated position using an automated sphygmomanometer (M3A, EDAN Instruments, INC., Shenzhen, China) placed on the subject’s right arm. The reading with lowest SBP was recorded for analysis of BP. Mean arterial pressure (MAP) was estimated by the equation: (2/3)*diastolic pressure (DBP) + (1/3)*systolic pressure (SBP). Subjects were requested to fast for a minimum of 12 hours and to avoid a high-fat diet or alcohol consumption for at least 24 hours prior to phlebotomy. Freshly voided urine samples were used for urinary albumin and creatinine measures by a biochemical test (UniCel^®^DxC 800 MA&CREA. Reagent). Spot urine albumin-creatinine ratios (ACR) were calculated for all participants. Venous blood samples were obtained at 5:308:00–11:00 am and stored in a 4°C refrigerator prior to analysis in the hospital laboratory. Clinical chemistry workup included fasting plasma glucose (FPG), total cholesterol (TC), triglycerides (TG), high-density lipoprotein cholesterol (HDL-C), serum creatinine (sCr) and urine creatinine (uCr), were measured by a biochemical auto-analyzer (DxC 800,Beckman Coulter UniCel^®^ DxC SYNCHRON^®^, Ireland). Blood tests were carried out in accordance with the hospital’s laboratory SOP that was accredited by the College of American Pathologists (CAP).

Serum Cr was used to estimate glomerular filtration rate (eGFR) using the Modification of Diet in Renal Disease (MDRD) Study equation in Nepalese participants, an alternative equation for estimating GFR in participants from the Chinese and Mongolian centers [29]: eGFR4 (mL/min per 1.73 m^2^) = 175 × SCr^-1.234^ × Age^-0.179^ × 0.79 (if female). On the basis of ACR values, albuminuria was classified according to urinary secretion of albumin into normoalbuminuria (ACR < 30 mg/g Cr), MAU (ACR: 30–299 mg/g Cr), and macroalbuminuria (ACR > 300mg/g Cr) according to the cutoffs recommended [30]. Hypertension was defined as systolic BP ≥ 140 mm Hg or diastolic BP ≥ 90 mm Hg, or receiving antihypertensive medication. Diabetes was defined as fasting blood glucose ≥ 7.0 mmol/L.

MetS was defined according to the National Cholesterol Education Program Adult Treatment Panel III (NCEP-ATP III) criteria [21] with modification on WC cutoff for Asian population suggested by the 1998 World Health Organization Asian Pacific Guideline [22]. MetS was defined as having three or more of the following factors: (1) Central obesity: WC ≥90 cm in men and ≥80 cm in women; (2) High BP: BP ≥130/85 mmHg or taking antihypertensive drugs; (3) High TG: TG ≥ 1.69 mmol/L (150 mg/dL); (4) Low HDL-C: HDL-C, ≤1.03 mmol/L (40 mg/ dL) in men and ≤1.29 mmol/L (50 mg/dL) in women; (5) Hyperglycemia: FPG ≥ 6.1 mmol/L (110 mg/dL) or taking anti-diabetic medication.

### Statistical analyses

Statistical analyses were performed using SPSS 16.0 statistics software (SPSS Inc., Chicago, IL). Continuous data were presented as mean and standard deviation (SD). The differences in continuous data were compared using independent two-sample t-test. One-way ANOVA together with Scheffe post-hoc tests were applied for comparing differences among groups. The associations between categorical variables presented as count and percentage were evaluated with Chi-square tests. The associations between ACR and metabolic risk factors were evaluated by linear regression analyses. Univariate and multivariable logistic regression models were performed to yield the odds ratios (ORs) of variables for the presence of albuminuria.

Receiver operating characteristic (ROC) analysis and the area under the ROC curve (AUC) were used to assess the accuracy and discriminatory ability of MS risk factors in detecting albuminuria. The optimal cut-point for each MS risk factor associated with CKD was established based on the AUC and Youden’s index. All statistical assessments were two-sided, and a *p* value <0.05 was considered statistically significant.

## Results

### Characteristics of the study population

Table 1 shows the clinical characteristics of the study population. In total, 18,384 subjects were analyzed for the study and 56.2% of the participants were males (n = 10,331). No significant difference was detected for age, ACR, and the percentages of subjects using anti-hypertensive or anti-lipid drugs between males and females. The frequency of anti-hypertensive or anti-lipid drug use was low, only accounting for 0.72% in males and 0.68% in females, respectively. Males had significantly higher levels for all variables, including BMI, WC, WHtR, SBP, DBP, MAP, TC, TG, TG/HDL-C ratio, and Cr (all *p* values < 0.0001) except HDL-C. Based on the findings of gender differences, the following analyses were performed separately for males and females.

**Table 1:** General clinical and metabolic characteristics of the study subjects.

Characteristics	Males	Females	p-value
	(n = 10,331)	(n = 8,053)	
Age (y/o)	42.9 ± 11.0	43.0 ± 11.5	0.824
BMI	24.5 ± 3.3	22.7 ± 27.5	0.000
WC (cm)	88.8 ± 8.9	77.1 ± 9.1	0.000
WHtR	0.51 ± 0.05	0.49 ± 0.08	0.000
SBP (mm Hg)	123.6 ± 16.4	115.2 ± 18.7	0.000
DBP (mm Hg)	76.7 ± 10.9	68.9 ± 10.5	0.000
MAP (mmHg)	92.3 ± 12.1	84.4 ± 12.5	0.000
FPG (mmol/L)	5.43 ± 1.36	5.20 ± 0.93	0.000
TC (mmol/L)	5.20 ± 0.97	4.96 ± 0.96	0.000
TG (mmol/L)	1.74 ± 1.83	1.02 ± 0.92	0.000
HDL-C (mmol/L)	1.18 ± 0.27	1.42 ± 0.31	0.000
TG/HDL-C	1.67 ± 2.56	0.81 ± 1.11	0.000
Cr (umol/L)	82.1 ± 12.9	57.9 ± 9.9	0.000
eGFR (ml/min/1.73 m^2^)	105.9 ± 26.1	155.0 ± 37.5	0.000
ACR (mg/g Cr)	11.0 ± 64.1	12.2 ±72.1	0.232
Anti-hypertension drug (%)	0.72 (n = 74)	0.68 (n = 55)	0.826
Anti-lipid drug (%)	0.039 (n = 4)	0.012 (n = 1)	0.379

Values are expresses as means ± SD

BMI, body mass index; WC, waist circumference; WHtR, waist-to-height ratio; SBP, systolic blood pressure; DBP, diastolic blood pressure; MAP, mean arterial pressure; FPG, fasting plasma glucose; TC, total cholesterol; TG, triglycerides; HDL-C, high-density lipoprotein cholesterol; Cr, creatinine; eGFR, estimated glomerular filtration rate; ACR, albumin-creatinine ratio.

The p value was obtained by chi-square or ANOVA.

Table 2 presents the characteristics of male participants according to the albuminuria status. There were significant differences between all listed variables and albuminuria status except anti-lipid drug use (all *p* values <0.001). The prevalence rates of albuminuria were 7.6% in males and 4.2% in females, giving an average of 6.1% (1,121/18,384) of the total population, in which MAU and macroalbuminuria accounted for 5.5% and 0.6%. Clear relationships between increasing albuminuria levels with SBP, FPG, Cr, and ACR were observed. Similar observations were detected for females, showing significant differences across all variables (Table 3); however, some were not consistent with the findings for males. Significant relationships between increasing albuminuria levels were observed for BMI, WC, SBP, Cr, and ACR.

**Table 2:** Clinical and metbolic characteristics according to albuminuria status for males (n = 10,331).

Characteristics	A (n = 9,552)	B (n = 699)	C (n = 80)	p-value^a^
	Normal	Microalbuminuria	Macroalbuminuria	
	<30 mg/day	30–300 mg/day	> 300 mg/day	
Age (y/o)	42.7 ± 10.9	45.1 ± 11.9†	46.2 ± 13.0‡	0.000
No. (%)	9,552 (92.5)	699 (6.8)	80 (0.8)	0.000^b^
BMI	24.3 ± 3.3	25.9 ± 4.0†	25.6 ± 4.0 ‡	0.000
Waist circumstance (cm)	86.5 ± 8.8	90.9 ± 9.6†	90.7 ± 10.0‡	0.000
Waist-to-height ratio	0.51 ± 0.05	0.54 ± 0.06†	0.54 ±0.06‡	0.000
SBP (mm Hg)	122.6 ± 15.7	134.5 ± 19.3†	139.1 ± 25.4‡§	0.000
DBP (mm Hg)	76.0 ± 10.4	84.7 ± 13.5†	87.1 ± 19.1‡	0.000
Mean arterial pressure (mmHg)	91.6 ± 11.5	101.3 ± 14.7†	104.5 ± 20.4‡	0.000
Fasting glucose (mmol/L)	5.34 ±1.10	6.46 ± 2.72†	7.23 ± 3.86‡§	0.000
Total cholesterol (mmol/L)	5.19 ± 0.95	5.33 ± 1.17†	5.26 ± 1.14	0.001
Triglycerides (mmol/L)	1.68 ± 1.52	2.51 ± 4.07†	2.37 ± 2.48‡	0.000
HDL cholesterol (mmol/L)	1.19 ± 0.27	1.13 ± 0.26†	1.16 ± 0.27	0.000
TG/HDL-C	1.60 ± 2.23	2.51 ± 5.31†	2.20 ± 2.22	0.000
Cr (umol/L)	81.8 ± 11.3	83.4 ± 18.4†	96.9 ± 54.0‡§	0.000
eGFR (ml/min/1.73 m^2^)	105.9 ± 25.8	106.3 ± 29.5	95.2 ± 31.1‡§	0.001
ACR (mg/g Cr)	4.54 ± 4.96	43.4 ± 44.2†	496.1 ±514.6‡§	0.000
anti-hypertension drug (%)	59 (79.7)	14 (18.9)	1 (1.4)	0.006^b^
anti-lipid drug (%)	4 (100.0)	0 (0.0)	0 (0.0)	0.982^b^

BMI, body mass index; WC, waist circumference; WHtR, waist-to-height ratio; SBP, systolic blood pressure; DBP, diastolic blood pressure; FPG, fasting plasma glucose; TC, total cholesterol; TG, triglycerides; HDL-C, high-density lipoprotein cholesterol; Cr, creatinine; eGFR, estimated glomerular filtration rate; ACR, albumin-creatinine ratio

^a^One way ANOVA, post hoc: Scheffe test

^b^Chi-square test

†Significant difference between A and B, p < 0.05

‡Significant difference between A and C, p < 0.05

§Significant difference between B and C, p < 0.05

**Table 3:** Clinical and metabolic characteristics according to albuminuria status for females (n = 8,053).

Characteristics	Normalbuimuria (n = 7,711) <30 mg/day	Microalbuminuria (n = 316) 30–300 mg/day	Macroalbuminuria (n = 26)> 300 mg/day	p-value^a^
Age (y/o)	42.8 ± 11.4	45.1 ± 11.9†	46.2 ± 13.0‡	0.000
No. (%)	7711 (95.8)	316 (3.9)	26 (0.3)	0.000^b^
BMI	22.3 ± 3.2	24.1 ± 4.5†	26.4 ± 5.2 ‡§	0.000
WC (cm)	76.9 ± 8.9	81.9 ± 11.4†	87.1 ± 12.5‡§	0.000
WHtR	0.49 ± 0.08	0.52 ± 0.08†	0.55 ±0.08‡	0.000
SBP (mm Hg)	114.6 ± 18.0	131.0 ± 25.4†	140.4 ± 24.9‡§	0.000
DBP (mm Hg)	68.6 ± 10.2	76.2 ± 13.5†	80.6 ± 14.5‡	0.000
MAP (mmHg)	83.9 ± 12.0	94.4 ± 16.5†	100.5 ± 16.4‡	0.000
FPG (mmol/L)	5.18 ± 0.82	5.88 ± 2.20†	5.88 ± 1.34‡	0.000
TC (mmol/L)	4.96 ± 0.95	5.07 ± 0.93†	5.43 ± 1.78‡	0.005
TG (mmol/L)	1.01 ± 0.91	1.34 ± 1.04†	1.54 ± 0.84‡	0.000
HDL-C (mmol/L)	1.42 ± 0.31	1.34 ± 0.30†	1.34 ± 0.33	0.000
TG/HDL-C	0.79 ± 1.10	1.14 ± 1.14†	1.24 ± 0.84	0.000
Cr (umol/L)	57.8 ± 9.2	59.2 ± 11.5†	74.9 ± 61.7‡§	0.000
eGFR (ml/min/1.73 m^2^)	155.3 ± 37.4	149.6 ± 39.4	141.9 ± 62.1	0.006
ACR (mg/g Cr)	7.30 ± 8.87	79.8 ± 93.8†	679.7 ± 1038.0‡§	0.000
Anti-hypertension drug (%)	46 (83.6)	9 (16.4)	0 (0.0)	0.000^b^
Anti-lipid drug (%)	0 (0.0)	1 (100.0)	0 (0.0)	0.000^b^

BMI, body mass index; WC, waist circumference; WHtR, waist-to-height ratio; SBP, systolic blood pressure; DBP, diastolic blood pressure; FPG, fasting plasma glucose; TC, total cholesterol; TG, triglycerides; HDL-C, high-density lipoprotein cholesterol; Cr, creatinine; eGFR, estimated glomerular filtration rate; ACR, albumin-creatinine ratio

^a^One way ANOVA, post hoc: Scheffe test

^b^Chi-square test for No. (%), AHD% and ALD%

†Significant difference between A and B, p < 0.05.

‡Significant difference between A and C, p < 0.05.

§Significant difference between B and C, p < 0.05.

The predicted influences of metabolic risk factors on ACR by linear regression analysis are presented in Table 4. For all participants, factors that displayed significant positive or negative associations with ACR included WC, WHtR, SBP, DBP, FPG, TC, and TG. For males, age, SBP, DBP, FPG, TC, TG, and TG/HDL-C ratio appeared significant relationships with ACR; however, only SBP, FPG, and TG were significant in females.

**Table 4:** Association of albumin-creatinine ratio (ACR) with metabolic risk factors by linear regression analysis.

	Coefficient (95% CI)		
Characteristic	All (18,384)	Male (n = 10,331)	Female (n = 8,053)
Age (y/o)	0.014 (−0.110–0.180)	0.022 (0.004–0.250)*	−0.024 (−0.327–0.029)
BMI	0.009 (−0.405–0.745)	−0.028 (−1.376–0.296)	0.009 (−0.708–1.108)
WC (cm)	−0.058 (−0.606 - −0.170)***	−0.022 (−0.576–0.257)	0.003 (−0.351–0.394)
WHtR	0.026 (1.939–50.208)*	0.022 (−48.530–101.764)	0.005 (−24.386–32.451)
SBP (mmHg)	0.078 (0.201–0.387)***	0.079 (0.184 −0.435)***	0.077 (0.150 −0.443)***
DBP (mmHg)	0.035 (0.064–0.355)**	0.048 (0.091–0.472)**	0.030 (−0.029–0.445)
FPG (mmol/L)	0.091 (4.331–6.053)***	0.126 (5.024–6.900)***	0.031 (0.570–4.243)**
TC (mmol/L)	−0.019 (−2.510 - −0.156)*	−0.027 (−3.259 - −0.353)*	0.005 (−1.668–2.418)
TG (mmol/L)	0.102 (2.494–6.440)***	0.122 (2.269–6.245)***	0.117 (1.849–16.585)*
HDL-C (mmol/L)	0.014 (−0.767–7.030)	0.004 (−4.380–6.419)	−0.003 (−7.408–5.482)
TG/HDL-C	−0.067 (−3.552 - −0.754)	−0.081 (−3.408 - −0.643)**	−0.083 (−11.353–0.573)

BMI, body mass index; WC, waist circumference; WHtR, waist-to-height ratio; SBP, systolic blood pressure; DBP, diastolic blood pressure; FPG, fasting plasma glucose; TC, total cholesterol; TG, triglycerides; HDL-C, high-density lipoprotein cholesterol.

*p < 0.05

** *p* < 0.01

*** *p* < 0.001.

### Factors associated with albuminuria

Table 5 shows the results of univariate logistic regression analysis to identify the risk factors potentially associated with the presence of albuminuria. These factors included various categories or levels of age, gender, WC, WHtR, hypertension, FPG, and TG/HDL-C. The risk of albuminuria was significantly higher for the elderly compared to younger participants, with a corresponding age-related increase. Males had also a nearly 2-fold higher risk than females (OR = 1.86; 95% CI: 1.63–2.13). Compared to those with a WC < 80 cm, a 10-cm increase in WC was associated with increased ORs of 1.75 (95%CI: 1.48–2.06), 3.80 (95% CI: 3.21–4.49), 6.25 (95%CI: 4.88–8.01), and 9.87 (95%CI: 5.87–16.58) within 80–90, 90–100, 10–110, and > 100 cm categories, respectively. A 0.1 increase in TG/HDL-C ratio was also related to corresponding increased ORs of 2.49 (95%CI: 2.17–2.86), 5.63 (95%CI: 4.45–7.12), and 11.23 (95%CI: 4.66–27.08) in those had a ratio of 0.5–0.6, 0.6–0.7, and > 0.7 than the participants with 0.5. Hypertension was associated with a 4-fold higher risk of albuminuria (OR = 4.05, 95%CI: 3.30–4.96) than non-hypertensive participants. In addition, increased risk was corresponding to FPG levels, giving ORs of 2.62 (95%CI: 2.10–3.28) and 6.73 (95%CI: 5.60–8.80) for those identified with IFG and DM. The level of risk also significantly increased with the higher TG/HDL-C ratio categories, with per 0.2 increases compared to 0.4.

**Table 5:** Association between risk factors and the presence of albuminuria (>30 mg/day) by univariate logistic regression analysis.

Category	Odds ratio (95%CI)	*p*-value
Age (y/o)		
15~30	Reference	
30–45	1.049 (0.843–1.306)	0.666
45~60	1.364 (1.086–1.712)	0.008
> 60	2.338 (1.807–3.026)	0.000
Gender		
Women	Reference	
Men	1.863 (1.631–2.129)	0.000
WC (cm)		
< 80	Reference	
80–90	1.745 (1.477–2.062)	0.000
90–100	3.795 (3.210–4.486)	0.000
100–110	6.253 (4.880–8.013)	0.000
> 110	9.867 (5.873–16.579)	0.000
WHtR		
<0.5	Reference	
0.5–0.6	2.490 (2.171–2.856)	0.000
0.6–0.7	5.628 (4.446–7.124)	0.000
> 0.7	11.232 (4.659–27.081)	0.000
Hypertension		
No	Reference	
Yes	4.048 (3.302–4.962)	0.000
FPG		
Normal	Reference	
IFG	2.623 (2.096–3.283)	0.000
DM	6.730 (5.604–8.083)	0.000
TG/HDL-C		
<0.4	Reference	
0.4–0.6	1.445 (1.078-.937)	0.014
0.6–0.8	1.772 (1.321–2.377)	0.000
0.8–1.0	1.789 (1.312–2.440)	0.000
1.0–1.2	2.422 (1.774–3.306)	0.000
1.2–1.4	2.846 (2.072–3.908)	0.000
1.4–1.6	2.859 (2.043–4.000)	0.000
1.6–2.0	3.426 (2.405–4.381)	0.000
2.0–2.5	3.469 (2.532–4.752)	0.000
2.5–3.0	4.158 (2.942–5.878)	0.000
>3.0	5.773 (4.361–7.642)	0.000

WC, waist circumference; WHtR, waist-to-height ratio; Hypertension: BP≥130/85 mmHg or current treatment; FPG, fasting plasma glucose; IFG: impaired fasting plasma glucose, 6.1–7.0 mmole/L; DM; fasting plasma glucose > 7.0 mmole/L.

In multiple logistic regression analysis, the factors significantly associated with the presence of MAU were WC > 90cm, WHtR at 0.6–0.7, hypertension, FPG > 6.1 mmole/L, and TG/HDL-C ratio > 1.6 (Table 6). With various ranges, these factors similarly increased the risk of developing MAU, except male gender. However, older age (>60 years) and WHtR greater than 0.7 did not maintain statistical significance associated with increased risk.

**Table 6:** Multiple logistic regression analysis of risk factors for developing microalbuminuria and albuminuria.

	Microalbuminuria (30–300 mg/day)		Albuminuria (> 30 mg/day)	
Category	Odds ratio (95%CI)	*p*-value	Odds ratio (95%CI)	*p*-value
Age (y/o)				
15~30	Reference		Reference	
30~45	0.760 (0.602–0.959)	0.021	0.755 (0.601–0.948)	0.015
45~60	0.708 (0.549–0.911)	0.007	0.721 (0.564–0.923)	0.009
> 60	1.039 (0.774–1.395)	0.797	1.069 (0.803–1.423)	0.648
Gender				
Women	Reference		Reference	
Men	1.161 (0.977–1.380)	0.089	1.196 (1.011–1.415)	0.037
WC (cm)				
< 80	Reference		Reference	
80–90	1.053 (0.822–1.348)	0.684	1.047 (0.823–1.331)	0.710
90–100	1.750 (0.822–1.348)	0.000	1.715 (1.272–2.312)	0.000
100–110	2.161 (1.415–3.300)	0.000	2.301 (1.529–3.466)	0.000
> 110	2.579 (1.211–5.491)	0.014	2.812 (1.360–5.814)	0.005
WHtR				
<0.5	Reference		Reference	
0.5–0.6	1.253 (0.993–1.581)	0.057	1.252 (0.999–1.570)	0.051
0.6–0.7	1.574 (1.054–2.351)	0.027	1.544 (1.045–2.282)	0.029
> 0.7	2.098 (0.640–6.884)	0.221	1.883 (0.580–6.119)	0.293
Hypertension				
No	Reference		Reference	
Yes	2.682 (2.129–3.379)	0.000	2.548 (2.032–3.196)	0.000
FPG				
Normal	Reference		Reference	
IFG	1.721 (1.349–2.195)	0.000	1.647 (1.295–2.094)	0.000
DM	3.881 (3.144–4.789)	0.000	4.133 (3.378–5.057)	0.000
TG/HDL-C				
<0.4	Reference		Reference	
0.4–0.6	1.186 (0.874–1.609)	0.274	1.214 (0.898–1.641)	0.208
0.6–0.8	1.203 (0.877–1.650)	0.252	1.291(0.948–1.760)	0.105
0.8–1.0	1.099 (0.785–1.540)	0.582	1.123 (0.806–1.565)	0.493
1.0–1.2	1.334 (0.943–1.887)	0.104	1.436 (1.024–2.013)	0.036
1.2–1.4	1.574 (1.108–2.235)	0.011	1.534 (1.083–2.172)	0.016
1.4–1.6	1.384 (0.949–2.019)	0.092	1.430 (0.987–2.071)	0.059
1.6–2.0	1.553 (1.104–2.185)	0.012	1.598 (1.143–2.236)	0.006
2.0–2.5	1.581 (1.104–2.264)	0.012	1.533 (1.074–2.189)	0.019
2.5–3.0	1.662 (1.113–2.481)	0.013	1.805 (1.225–2.660)	0.003
>3.0	2.307 (1.655–3.216)	0.000	2.386 (2.721–3.307)	0.000

WC, waist circumference; WHtR, waist-to-height ratio; HDL-C, high-density lipoprotein cholesterol; TG, total cholesterol; Hypertension: BP≥130/85 mmHg or current treatment; FPG, fasting plasma glucose; IFG: impaired fasting plasma glucose, 6.1–7.0 mmole/L; DM; fasting plasma glucose > 7.0 mmole/L.

### Comparison of the predictive accuracy of factors associated with the presence of albuminuria

The predictive accuracy of variables associated with the presence albuminuria with statistical significance in logistic regression analyses were further compared. Table 7 presents the association of AUC values of WC, WHtR, MAP, FPG, and TG/THL-C with the presence albuminuria in both males and females. According to a rough guide for classifying the accuracy of diagnostic test, although an AUC of 0.6–0.7 only represent a fair (or not good) test, it still provides sufficient ability to discriminate the presence of albuminuria. The optimal cut-off values, according to Youden’s index, of variables were all higher in males than females. For the presence of albuminuria, cut-offs of WC were 90.8 vs. 80.0 cm (males vs. females), WHtR were 0.53 vs. 0.52 (males vs. females), MAP were 97.9 vs. 91.9 mmHg (males vs. females), FPG were 5.40 vs. 5.28 mmole/L (males vs. females), and TG/HDL-C were 1.13 vs. 1.08 (males vs. females), respectively.

**Table 7:** The areas under the ROC curve (AUC), sensitivity and specificity by the optimized cut-off points for metabolic risk factors associated with the presence of albuminuria (> 30mg/day).

Risk factor	AUC (95% CI)	Cut-off according to Youden's index	Sensitivity (%)	Specificity (%)
**Male (n = 10,343)**				
WC (cm)	0.643 (0.622–0.664)	90.8	54.2	68.8
WHtR	0.644 (0.623–0.665)	0.53	61.6	61.6
MAP (mmHg)	0.695 (0.675–0.715)	97.9	56.7	72.5
FPG (mmole/L)	0.674 (0.654–0.695)	5.40	54.8	70.0
TG/HDL-C ratio	0.614 (0.593–0.635)	1.13	67.3	49.0
**Female (n = 8,091)**				
WC (cm)	0.646 (0.613–0.680)	80.75	56.7	68.0
WHtR	0.649 (0.615–0.682)	0.52	56.4	70.4
MAP (mmHg)	0.706 (0.676–0.737)	91.9	55.6	76.9
FPG (mmole/L)	0.622 (0.589–0.656)	5.28	51.5	68.8
TG/HDL-C ratio	0.629 (0.597–0.660)	1.08	40.4	80.9

WC, waist circumference; WHtR, waist-to-height ratio; MAP, mean arterial pressure; FPG, fasting plasma glucose; HDL-C, high-density lipoprotein cholesterol; TG, total cholesterol.

## Discussion

Results of this study indicated that the prevalence of MAU and macroalbuminuria were of 5.5% and 0.6% in Chinese adult population. Age, male gender, WC, WHtR, hypertension, FPG, and TG/HDL-C were significantly associated with the presence of albuminuria in univariate logistic regression analyses. WC > 90cm, WHtR 0.6–0.7, hypertension, FPG > 6.1 mmole/L, and TG/HDL-C ratio > 1.6 maintained statistical significance in multiple logistic regression analysis, but not age or male gender. Higher WHtR was correlated to increased risk of MAU; however, statistical significance disappeared when WHtR was greater than 0.7.

The prevalence of MAU in this study was higher than Japanese [31], similar to Korean [32], slightly lower than those in Australia (6.0%) [33], Europe (7%) [34], and in the US (7.8%)[35]. It is likely that similar results to Korean [32] are due to similarity in the mean ages between populations (45.6 vs. 43.0 years), and which were younger than other studies. Greater age was reported to be associated with MAU and macroalbuminuria in Korean [32]; nevertheless, this study did not have a similar finding. Furthermore, consistent with the studies in Korea[32], US [36] and Japan[37], we did not find that males had an increased risk of developing albuminuria than females in multiple logistic regression analysis. It is likely that ethnic difference may also contribute to variations in the presence of albuminuria.

Despite the associations between MetS and MAU were consistent in previous studies, associations between individual MetS components and MAU were controversial. For example, FPG and BP were consistently found to be two main risk factors associated with MAU [38], as was also clearly evident from the present study; however, inconsistencies were found for abdominal obesity and HDL-C and TG levels [12, 15, 25]. In addition, our results are in line with some previous reports that demonstrated an association of MAU with CVD risk factors, including hypertension, hypertriglyceridemia, impaired fasting glucose, and diabetes [39, 40].

Although the mechanisms of the association between MetS and albuminuria are not yet fully understood, increased WC [41] and TG/HDL-C ratio in addition to hypertension and diabetes could possibly be responsible for increased endothelial permeability and intraglomerular capillary pressure and consequent development of atherosclerosis and glomerulopath. Even though the underlying mechanism of MAU mediates the pathology of CKD, CVD and diabetes is not entirely clarified, the presence of MAU reflects progressive endothelial and vascular dysfunction[42, 43]. Additionally, The percentages of individuals taking antihypertensive drugs were very low (approximately 0.7%) in this study population, which decreased the cofounding effect of various antihypertensive agents [44] on the occurrence of MAU in this study.

Additionally, the discriminatory values of WC, WHtR, MAP, FPG, and TG/HDL-C for the presence of albuminuria was not ideal, as all areas of under ROC were less than 0.7, considering that cutoff levels are defined depending on the cost-effectiveness of screening, they can be an useful tool in an attempt to prevent CVD and CKD in individuals with increased renal and cardiovascular risks, at least for Chinese adults. Investigation with dedicated optimal design for other population is recommended.

As a previous study indicated that low-grade albuminuria was significantly associated with the increasing prevalence of MetS and its components in middle-aged and elderly Chinese population[28], early intervention can be effective on delaying the natural progression of chronic kidney disease and diabetic nephropathy. A public health approach without aggressive medical intervention that aimed at lifestyle modification may also benefit general population.

Our study corroborates the findings in recent reports demonstrating that all components of MetS were significantly associated with albuminuria [27] [45], and extends the available knowledge, it shows the optimal cutoffs for each component of MetS associated with the presence of MAU in male and female Chinese. Further investigations may be required to determine the underlying mechanisms link to the gender differences in the cut-off points associated with the presence of albuminuria in practice.

### Limitations

The strengths of our study include the use of a population- based sample and adopted the MDRD study equation modified for Chinese. However, there were some limitations in this study. Firstly, this study was cross-sectional and lack of long-term follow-up, causal relationship between risk factors and albuminuria could not be established because the potential effect of variability related to metabolic control might be underestimated Secondly, a selection bias might have been introduced due to the study subjects were included from our health checkup program. Self-reported histories might have also caused misclassification. Thirdly, a single urine ACR result was assessed in this analysis, which could result in misleading classifications of albuminuria [46]. Fourthly, some potential confounding factors, such as Chinese herbal medicine use [47], salt intake [31], other medications or comorbidities could not be ruled out.

## Conclusions

The present study shows that MetS and all its components were associated with the presence of MAU in a general Chinese population. Differences in the optimal cutoffs for each component of MetS associated with the presence of MAU were detected between males and females. Thus, assessment of MetS risk factors can open a window of opportunity for early intervention to decrease the effect of a deterioration of metabolic control and subsequent albuminuria.
